# Effects of Extender Type, Storage Time, and Temperature on Bull Semen Parameters

**DOI:** 10.3390/biology10070630

**Published:** 2021-07-07

**Authors:** Aitor Fernandez-Novo, Sergio Santos-Lopez, Clara Barrajon-Masa, Patricia Mozas, Eduardo de Mercado, Elisa Caceres, Aizic Garrafa, Juan Vicente Gonzalez-Martin, Natividad Perez-Villalobos, Agustin Oliet, Susana Astiz, Sonia Salome Perez-Garnelo

**Affiliations:** 1 Department of Veterinary Medicine, School of Biomedical and Health Sciences, Universidad Europea de Madrid, C/Tajo s/n, Villaviciosa de Odón, 28670 Madrid, Spain; aitor.fernandez@universidadeuropea.es; 2 Animal Production Department, Veterinary Faculty, Complutense University of Madrid, Puerta de Hierro Avenue s/n, 28040 Madrid, Spain; sesantos@ucm.es; 3 National Centre of Selection and Animal Reproduction (CENSYRA) of Colmenar Viejo, Ctra. Colmenar Viejo—Guadalix de la Sierra km 1, Guadalix de la Sierra, 28794 Madrid, Spain; clara.barrajon@madrid.org (C.B.-M.); patricia.mozas@madrid.org (P.M.); agustin.oliet@madrid.org (A.O.); 4 Animal Reproduction Department, National Institute of Agronomic Research (INIA), Puerta de Hierro Avenue s/n, 28040 Madrid, Spain; eduardo.mercado@inia.es (E.d.M.); astiz.susana@inia.es (S.A.); sgarnelo@inia.es (S.S.P.-G.); 5 Department of Animal Medicine and Surgery, Veterinary Faculty, Complutense University of Madrid, Puerta de Hierro Avenue s/n, 28040 Madrid, Spain; elicacer@ucm.es (E.C.); aizicbg@gmail.com (A.G.); juanvi@ucm.es (J.V.G.-M.)

**Keywords:** BBSE, short-term storage, semen extender, sperm viability, sperm motility, microbiological load

## Abstract

**Simple Summary:**

The bull accounts for a great part of the economic value of beef herds managed with natural service. Therefore, the bull breeding soundness evaluation (BBSE) becomes essential in such cattle herds. Part of BBSE is the semen-quality evaluation (classical and kinetic parameters), which can be performed in situ (by trained practitioners) or at laboratories, with semen being short-term stored and shipped. The extender used, storage temperature, and duration may affect its quality. Thus, our aim was to explore this in order to define the best conditions to preserve seminal quality, to be reliably assessed afterwards. Semen quality was preserved for up to 4–6 h post-ejaculation, except for INRA96^®^ (IMV Technologies, L’Aigle, France) at 5 °C. Motility decreased from 4 to 6 h up to 24 h, with the best values obtained with BIOXcell^®^ (IMV Technologies, L’Aigle, France) at 5 °C. Microbiological load was stable over time with AndroMed^®^ (Minitub Ibérica SL, Tarragona, Spain) and BIOXcell^®^, and increased at room temperature with INRA96^®^. Therefore, we suggest to evaluate seminal quality as soon as possible. However, using AndroMed^®^ and BIOXcell^®^, either at 5 °C or room temperature, semen can be reliably evaluated for up to 6 h, while INRA96^®^ can be used at room temperature. These results help to fix adequate protocols for short-term storage and shipment of bovine semen collected under field conditions.

**Abstract:**

Seminal parameters can be evaluated in situ, or samples can be delivered to a diagnostic centre. How storage conditions affect ejaculates up to evaluation is unclear. We assessed, in 25 commercial bulls electroejaculated in the field, the impact of time until evaluation (0–2 h, 4–6 h, and 24 h post-ejaculation), holding temperature (5 °C vs. room temperature), and extender (AndroMed^®^, BIOXcell^®^ or INRA96^®^) on semen quality. Acrosome integrity, sperm viability and morphology, CASA-total and progressive motility, pH, and colony-forming units were assessed. Semen quality was preserved for up to 4–6 h post-ejaculation, except for INRA96^®^ at 5 °C. Regardless of extender or temperature, motility decreased from 4 to 6 h up to 24 h, with the best values obtained with BIOXcell^®^ at 5 °C. pH differed from 4 to 6 h up to 24 h, acidifying when stored at room temperature. Microbiological load was stable over time with AndroMed^®^ and BIOXcell^®^, and increased at room temperature with INRA96^®^. Our results suggest that AndroMed^®^ and BIOXcell^®^ can preserve semen quality for up to 6 h, either at 5 °C or room temperature, while INRA96^®^ only at room temperature. These results help to fix adequate protocols for short-term storage and shipment of bovine semen collected under field conditions.

## 1. Introduction

The bull is very relevant to the economic value of the cattle herds with reproductive management based on natural service [1]. It has been documented that the bull can determine 80–85% of the reproductive efficiency of these herds [2]. It is considered that up to 20–40% of bulls are subfertile, and a few of them are completely infertile [3,4]. The bull breeding soundness evaluation (BBSE) allows the detection of subfertile and infertile bulls. Therefore, it is advisable that all mating bulls of herds are submitted to a BBSE yearly [5,6] or even more frequently, because of the effect of season on semen quality [7]. Bull fertility can be 5% higher in herds where a routine BBSE is annually implemented than in herds where it is implemented less often or not at all [8], owing to the early culling and substitution of subfertile bulls.

Several countries have developed regulated BBSE systems with standardized criteria to be applied in the field. These countries include the USA, which was the first to adopt a national BBSE guide (from the Society for Theriogenology (SFT)) [9], Canada [10], Australia [11], South Africa [12], and UK [13]). BBSE guidelines differ by country, reflecting the local characteristics of herds and farms. Nevertheless, in all cases, the methodology assesses sight, foot poise, locomotion, internal and external genital system, scrotal circumference, and reproductive bull behavior. Additionally, the ejaculate is evaluated in terms of classical and kinetic sperm parameters.

Several parameters to assess semen quality in the field have been described [14]. In general, sperm motility is considered the main indicator of semen quality and fertility [14,15], although it may miss-estimate fertility, with some studies evidencing a not consistent correlation between these two characteristics [16,17]. To obtain an objective and repeatable motility estimation, computer-assisted semen analysis (CASA) was developed [18,19]. CASA gives special emphasis to certain aspects of bull sperm motion, including the behavior of subpopulations of spermatozoa and the determination of kinetic parameters [14]. CASA should be supplemented with morphological examination of semen and determination of the percentage of morphologically normal spermatozoa [16].

In most of the countries, semen quality evaluation is performed in situ on the farm [9,10,11,12,13]. In contrast, the Australian Cattle Veterinarian Association recognizes only valid sperm morphology assessment issued by reference laboratories [11]. In this case, semen needs to be stored, transported, and evaluated after arrival at the laboratory. Similarly, in other countries where veterinarians rarely perform semen quality assessment themselves [20], semen needs to be stored for short or intermediate durations. In addition, short-term semen storage is essential to allow subsequent semen cryopreservation from highly valuable bulls [21] or to allow insemination with fresh bull semen [22,23]. It is also important for farms located far away from reference laboratories and for farms under extreme climate conditions [24]. Short-term semen storage is necessary on farms where veterinary practitioners begin semen evaluation in the field.

The conditions of semen storage may affect its quality, depending on the extender type used, storage temperature, and storage duration. The preserving ability of semen extenders under refrigeration temperatures depends on whether they include cryoprotectants, even during short-term storage lapses [25,26]. Penetrating cryoprotectant agents, such as glycerol, pass through the cell membrane and protect the cell from damage caused by slow freezing [25,27]. Non-penetrating cryoprotectant agents, including dextrose, dextran, and polyethylene glycol, among others [25], promote rapid cell dehydration to avoid the formation of ice crystals; they are often used together with penetrating cryoprotectant agents. Other substances can also act as cryoprotectants via various mechanisms, including milk proteins, albumin, and liposomes. These substances change the lipid composition of the cell membrane, increasing its permeability to penetrating cryoprotectant agents and providing greater resistance to temperature shock [15,25,27]. Studies of the efficacy of commercially available soy lecithin-based extenders have shown that extenders can affect intrinsic and extrinsic semen factors, including fertility, calving rate, and motility [28]. Apart from fertility, extenders can also influence motility [22].

Storage at room temperature for 72 h has been shown to affect motility depending on the extender used [22]. Several studies have focused on the influence of freezing and thawing on semen quality [29], but few studies have systematically examined how storage conditions affect key seminal parameters in short- and medium-stored semen, not cryopreserved.

Therefore, our aim in the present study was to mimic different field conditions of short-term storage (time, temperature, and extender: AndroMed**^®^** [30], BIOXcell**^®^** [31], and INRA96**^®^** [32]) of bull semen from collection at the farm until delivery to the laboratory and to measure the effects of that storage on semen quality based on sperm viability, morphology, motility, pH, and microbiological quality. Our results lead to recommendations for semen storage in the field.

## 2. Material and Methods

### 2.1. Animals and Herds

Bulls from 14 commercial beef cattle farms and 1 commercial dairy farm in central Spain were included in the study if, at the time of sperm collection, (a) they had been apart from the cow herd for more than 15 days; (b) their rectal temperature was <39.0 °C, in order to avoid possible diseases affecting seminal quality; (c) they were >24 months old; (d) testicular ultrasonography using a SIUI CTS 800**^®^** (Shantou Institute of Ultrasonic Instruments Co., Ltd., Guangdong, China) system with 7.5 MHz transrectal transducer revealed no abnormalities; and (e) their scrotal circumference was >34 cm, to avoid abnormally developed bulls. A total of 25 electroejaculates were collected from 25 different bulls (one per bull) from five breeds: Limousine (*n* = 10), Charolais (*n* = 9), Blonde D’Aquitania (*n* = 1), Holstein Friesian (*n* = 1), and Spanish Black Iberian Avileña (*n* = 1), as well as cross-breeds (*n* = 3; Avileña by Limousine; Charolais by Limousine, and Avileña by Charolais). Samples were obtained during the routine BBSE of these animals, following advised protocols, such that the study interventions were not considered experimentation on animals and no ethical approval was required. Animals were restrained in safety squeeze chutes when explored and electroejaculated, and procedures were performed in a way that maximised animal welfare and worker safety.

Before electroejaculation, bulls had been tested for bovine viral diarrhea and infectious bovine rhinotracheitis, as well as infection with *Tritrichomonas foetus*, *Campilobacter foetus*, and *Besnoitia besnoiti*. Animals had also been tested for all officially notifiable diseases specified by the European Union (tuberculosis, brucellosis, peripneumonia, and bovine leucosis).

### 2.2. Ejaculate Collection

Bull semen was sampled while animals were restrained in the farm chute. Preputial fur was cut, then the preputium was washed with 40–60 mL of prewarmed physiological saline solution (NaCl 0.9%, B. Braun**^®^**, B. Braun Medical, SA, Barcelona, Spain) and dried with sterile swabs. Afterwards, transrectal palpation was performed to remove the faeces and to stimulate the bull before inserting the transrectal probe (75 mm transrectal probe; Electroejaculator Pulsator IV**^®^**, Lane Manufacturing Inc., Denver, CO, USA). An automatic program applied alternating stimuli of increasing intensity until ejaculation. Semen samples were collected in sterile 15 mL Falcon tubes immersed in a 50 mL tube with prewarmed water (37 °C) to avoid temperature shock.

### 2.3. Semen Storage Conditions

Semen samples were subjected to different conditions that mimicked typical field situations. Different time windows between collection and evaluation were considered: 0–2 h (“<2 h”), mimicking immediate analyses; 4–6 h, mimicking car transportation; and 24 h, mimicking postal delivery. Three commercial extenders widely used in the field were tested: AndroMed**^®^** (Minitube, Tiefenbach, Germany), BIOXcell**^®^** (IMV Technologies, L’Aigle, France)—both lecithin-based extenders—and INRA96**^®^** (IMV Technologies). Two storage (or holding) temperatures were tested: refrigeration (5 °C), mimicking the use of standard refrigerators or the temperatures promised by shipping companies, or room temperature (RT, 23–25 °C).

**Figure 1:**
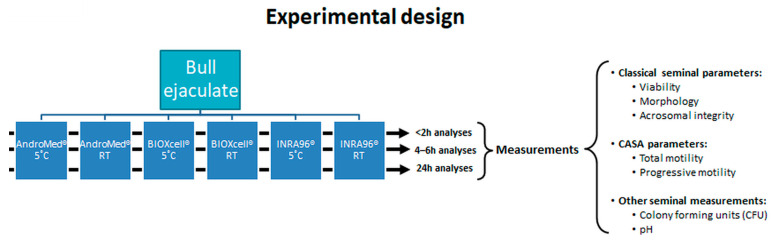
Experimental study design (3 × 3 × 2) involving three extenders, two holding temperatures, and three storage durations. Abbreviations: CASA = computer-assisted semen-analysis; RT = room temperature.

Immediately after semen collection, six 1:2 aliquots (1 mL ejaculate and 2 mL extender) were prepared—two for each extender. This dilution is the one advised by the diagnostic laboratories for practitioners, when sending semen samples for quality assessment within the BBS evaluations. To avoid thermal shock, the samples were immersed in a 50 mL Falcon tube containing water at 37 °C in order to reduce the temperature gradually. Three aliquots (one per extender) were kept at 5 °C and the other three at RT until evaluation. The samples stored at 5 °C were kept in a car-refrigerator with temperature control, and a data logger (176 H2; Testo, Barcelona, Spain) was used to measure the cooling ramp. Samples were identified as AndroMed**^®^** 5 °C (A5), AndroMed**^®^** room temperature (ART), BIOXcell**^®^** 5 °C (B5), BIOXcell**^®^** room temperature (BRT), INRA96**^®^** 5 °C (I5), and INRA96**^®^** room temperature (IRT), depending on extender and holding temperature (Figure 1).

From each aliquot, just before semen assessment, a sample of 400 µL was taken and immediately frozen in liquid nitrogen for microbiological analyses at a specialized laboratory (Labocor SL, Madrid, Spain).

### 2.4. Semen Quality Assessment

Semen aliquots were evaluated at a reference laboratory (National Centre of Selection and Animal Reproduction, CENSYRA; Madrid, Spain) at three time points after collection: <2 h, 4–6 h, and 24 h by the same specialist. Each aliquot was kept in the same conditions (temperature and extender) until its evaluation. Exact time intervals and temperature variations were monitored and recorded using a data logger.

Semen samples were evaluated in the laboratory in the following sequence: first AndroMed**^®^** samples, then BIOXcell**^®^** samples, and finally INRA96**^®^** samples. This sequence was followed for the room temperature and 5 °C samples. Assessments occurred about 5 min apart.

Sperm viability, normal sperm morphology, acrosomal status in live spermatozoa, CASA kinetic parameters, and pH were evaluated. Sperm viability and sperm morphology were assessed using eosin-nigrosin vital staining [33]. Percentages of live sperm, based on viability, and percentages of normal sperm, based on morphology analyses, were determined from 100 spermatozoa/slide in four slides/aliquot under bright-field microscopy (400×). Morphological abnormality was expressed as a percentage [10,11,13]. When ≥2 types of abnormality were found in the same spermatozoon, priority was assigned as follows: head abnormalities > intermediate piece abnormalities > tail abnormalities. In order to determine acrosomal status in live spermatozoa, the eosin-nigrosin slides were overstained with Giemsa [33,34]. This triple stain technique is capable of determining four categories of spermatozoa: live acrosome—intact (the key parameter assessed in our study), live acrosome—reacted or damaged, dead acrosome—intact, and dead acrosome—reacted or damaged. Percentages were calculated by counting 100 spermatozoa/slide in two slides/aliquot under bright-field microscopy (1000×).

We performed CASA with a system comprising a phase-contrast Nikon Eclipse Ci microscope (Nikon Imaging Japan Inc., Tokio, Japan) equipped with a heated stage, negative phase contrast 10× objective, and a digital camera (acA780-75 gc, Basler, Germany). The camera transmitted the images to a computer for analysis using Sperm Class Analyzer software (Microptic Automatic Diagnostic Systems SL, Barcelona, Spain). For analysis, aliquots (8 µL) of semen were added to a Spermtrack**^®^** 20 µm chamber pre-warmed at 37 °C. At least eight random fields were measured, such that at least 2000 sperm were assessed per sample. The software settings were those recommended by the manufacturer for analysis of bull semen motility. The cell identification area was 28–70 μm^2^. Sperm with curvilinear velocity (VCL) <20 μm/s were considered immotile; VCL 20–60 μm/s, slow; VCL 60–110 μm/s, medium; and VCL >110 μm/s, fast. Sperm with straightness >70 were considered progressively motile. Values of total and progressive motility were averaged over at least 2000 spermatozoa per sample.

Bacterial load was determined in terms of colony-forming units (CFUs) per mL using the standard plate count method at Labocor SL (Madrid, Spain). Semen samples were serially diluted in tryptone water. Inocula (50 µL) from each dilution were mixed thoroughly with molten agar (previously held in a water bath at 47 °C) and poured into sterile Petri dish plates. Separate plates were used for each dilution. The plates were incubated at 37 °C for 72 h. Colonies per plate were counted using a colony counter, and CFUs were calculated using the following formula: CFU/mL of sample = no. of CFU × dilution × 20.

The pH was assessed with indicator paper strips (Whatman**^®^** CS) capable of measuring from pH 1.8 to 9.7 in gradations of 0.2–0.3.

Based on the quality semen assessment, each bull was classified as suitable or not according to the most frequently used bull breeding soundness evaluation (BBSE) guidelines in Spain. These were the guidelines of the Society For Theriogenology (SFT) [9]; from UK [13] and the Spanish guidelines VART (“Valoración de la Aptitud Reproductiva de Sementales” or “Evaluation of Bull Reproductive Ability”) [20].

### 2.5. Statistical Analysis

All data were analysed using SPSS**^®^** version 25 (IBM, Armonk, NY, USA). Differences associated with *p*-values lower than or equal to 0.05 were considered significant. As all outcomes were continuous variables, data were reported as mean ± standard deviation (SD) in tables and text, but as mean ± standard error of the mean (SEM) in figures. CFUs were log10-transformed.

Outcomes from all variables (sperm viability in percentage; live acrosome—intact spermatozoa in percentage; sperm morphology in percentage, sperm total motility in percentage, progressive motility in percentage, microbiological quality in log10-CFU, and pH value) were analysed using a generalised linear mixed model including the factor bull as a random effect and kept in the model if having a statistically significant effect. All potential interactions among the studied factors were assessed (extender by holding temperature, extender by time, holding temperature by time, and extender by holding temperature by time). Intra-subject effect tests (Greenhouse–Geisser) and inter-subject effect tests were performed. Repeated-measures analysis of variance (ANOVA) was used to assess the significance of differences among aliquots stored for different periods. Potential pairwise correlations between variables were assessed using the Pearson correlation test separately for each of the storage durations (<2 h, 4–6 h, and 24 h), and intra-variable correlations across the three durations were also assessed.

## 3. Results

The bulls included in the study were 41.7 ± 23.3 months old (8 bulls < 24 months; 8 bulls aged between 24 and 48 months; 6 bulls aged between 48 and 72 months; and 3 bulls aged between 72 and 96 months) had a rectal temperature of 37.8 ± 0.51 °C at semen collection, and an average scrotal circumference of 40.3 ± 2.85 cm. Semen samples were obtained by the first electroejaculation for all bulls except for four bulls, which required two electro-cycles to collect the ejaculation. The average volume of the ejaculate was 6.7 ± 3.83 mL. The generalised linear mixed model revealed that the factor “bull” influenced all parameters (*p* < 0.01), so it was kept in the model.

Average time intervals between semen collection and evaluation were 1.3 ± 0.18 h for samples assessed at 0–2 h, 4.8 ± 0.01 h for samples assessed at 4–6 h, and 24.7 ± 0.49 h for those evaluated at 24 h. Each of these intervals did not differ significantly among samples mixed with different extenders (*p* > 0.1).

Actual temperature for the samples held at 5 °C was 6.8 ± 1.48 °C (0.4 ± 0.12 °C/min of cooling rate) at the first assessment time (<2 h). Afterwards, samples were kept in a laboratory refrigerator. The actual temperature for samples kept at room temperature was 24.5 ± 1.77 °C. No significant differences in holding temperature were observed among samples with different extenders (*p* > 0.1).

### 3.1. Semen Quality Based on Classical and Motility Parameters

**Figure 2:**
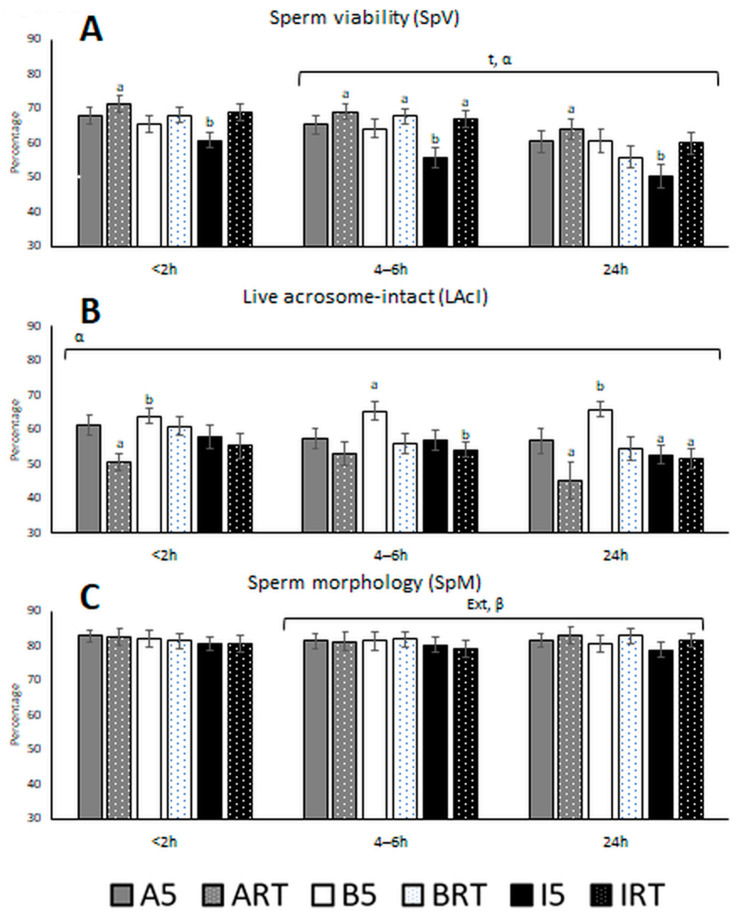
(**A**) Bull sperm viability (percentage), (**B**) live acrosome—intact spermatozoa (percentage), and (**C**) sperm morphology (percentage) after storage for three periods in the presence of three extenders at two temperatures. Abbreviations: A: AndroMed*^®^*; B: BIOXcell*^®^*; I: INRA96*^®^*; RT: room temperature; 5: refrigeration at 5 °C; t: time; Temp: temperature; Ext: extender; α: interaction temperature by extender; β: interaction temperature by time; γ: interaction extender by time; δ: triple interaction temperature by extender by time. Bars with different letters denote significantly different values (*p* < 0.05) among the six aliquots of the three extenders by two holding temperatures for each storage duration (analyses of variance (ANOVA)). Bars without letters were not significantly different. Square brackets over the bars highlight significant effects of the individual factor or its interactions with time (*p* < 0.05 from the generalised linear mixed model). The brackets cover the time window when the effect was significant, whether from <2 to 4–6 h, 4–6 to 24 h, or <2 to 24 h.

The results obtained for sperm viability, live acrosome—intact spermatozoa, and morphology are represented in Figure 2.

Sperm viability (SpV) decreased from 4–6 h to 24 h, independently of extender and holding temperature. The immediate assessment of sperm viability (<2 h post-ejaculation) or at 4–6 h resulted in SpV values without statistical differences. Analyses performed up to 24 h showed an important decrease in SpV, revealing that room temperature preserved better sperm viability than refrigeration temperature. The viability values ranged from 60.5% with INRA96*^®^* 5 °C to 71.3% with AndroMed*^®^* at RT; for all three durations, the highest values were observed with AndroMed*^®^* at RT (71.3, 69.0, and 64.0% at <2 h, 4–6 h, and 24 h, respectively), and the lowest with INRA96*^®^* at 5 °C (60.8, 56.7, and 50.4%, respectively).

The significant interaction of holding temperature with extender (*p* < 0.001) showed that extender performance varied with temperature: AndroMed*^®^* and INRA96*^®^* performed better at RT than at 5 °C, while the opposite was observed for BIOXcell*^®^*. INRA96*^®^* at 5 °C was associated with significantly smaller sperm viability than AndroMed*^®^* or BIOXcell*^®^*, regardless of holding temperature or duration (Figure 2A).

Global live acrosome—intact spermatozoa percentages were 63.8, 65.3, and 65.9% at <2 h, 4–6 h, and 24 h stored at 5 °C, respectively, and 61.0, 55.9, and 54.6 at <2 h, 4–6 h, and 24 h stored at room temperature, respectively. The determination of percentages of live acrosome—intact spermatozoa revealed a significant effect of the temperature by extender interaction (*p* < 0.001). Acrosomal integrity was best preserved with BIOXcell*^®^* at 5 °C at 24 h after collection. AndroMed*^®^* and INRA96*^®^* kept values stable over time when samples were kept at 5 °C, although these values were lower than those observed with BIOXcell*^®^*, regardless of storage duration. At RT, a significant decrease was observed from 4 to 24 h in samples preserved with AndroMed*^®^* or INRA96*^®^*.

Sperm morphology was affected by a significant interaction of temperature by time (*p* = 0.019) from 4–6 h to 24 h after semen collection. Average values of normal sperm morphology ranged between 79.1 and 83.1%. Detached head and distal reflex were the two most frequent morphological abnormalities observed (data non-shown).

**Figure 3:**
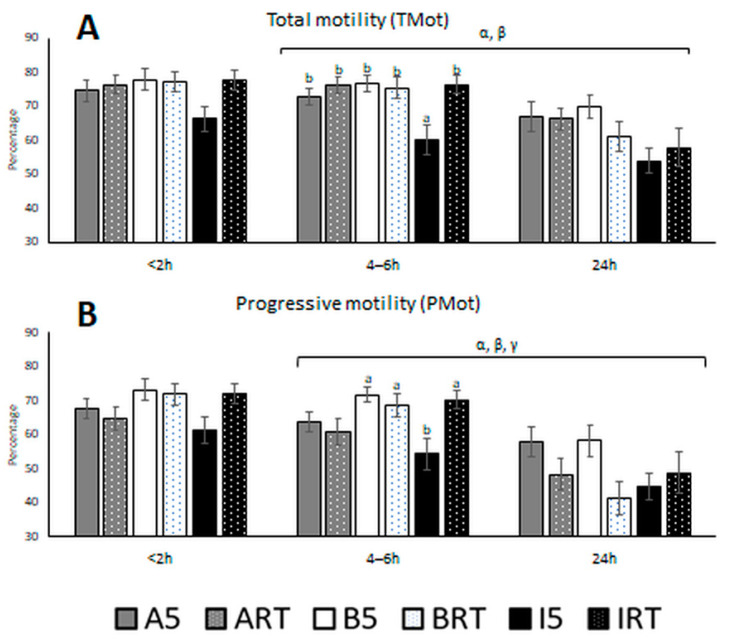
(**A**) Bull sperm total motility (percentage) and (**B**) progressive motility (percentage) after three storage durations in the presence of three semen extenders at two holding temperatures. Aliquot abbreviations: A: AndroMed*^®^*; B: BIOXcell*^®^*; I: INRA96*^®^*; RT: room temperature; 5: refrigeration at 5 °C. Factors abbreviations: t: time; Temp: temperature; Ex: extender; α: interaction temperature by extender; β: interaction temperature by time; γ: interaction extender by time; δ: interaction temperature by extender by time. Bars with different letters denote significantly different values (*p* < 0.05) among the six aliquots of the three extenders by two holding temperatures at each fixed time window (ANOVA). Bars without letters were not significantly different. Square bracket over the bars highlights a significant effect (*p* < 0.05; from generalized linear mixed model analyses) of the factor or factor interactions by time. The bracket covers the time window where the effect is significant (from <2 to 4–6 h, from 4–6 h to 24 h, or from <2 to 24 h).

Total and progressive motility determined in CASA are summarised in Figure 3.

Both motilities were affected by significant interactions of temperature with extender and time (Figure 3). With all extenders, a general decrease in motility was observed from 4–6 h to 24 h after semen collection, and this decrease was more pronounced at RT than at 5 °C. For the shortest storage (<2 h), the highest total motility was obtained with BIOXcell*^®^* when samples were stored at 5 °C (77.8 ± 14.7%). At 4–6 h, there were significant differences between the total motility of INRA96*^®^* samples at 5 °C and all other samples. Motility at 24 h was markedly lower than at earlier time points; the lecithin-based extenders AndroMed*^®^* and BIOXcell*^®^* preserved total motility better.

BIOXcell*^®^* gave better total motility at 5 °C than at RT, while INRA96*^®^* gave better motility at RT. Samples with AndroMed*^®^* showed better progressive motility at 5 °C. For all storage durations, INRA96*^®^* at 5 °C led to the lowest motility. At the shortest storage (<2 h), BIOXcell*^®^* samples at 5 °C showed the highest progressive motility (73.2 ± 15.2%). At 4–6 h, the progressive motility of INRA96*^®^* samples held at 5 °C (54.3 ± 22.12%) differed from that observed with BIOXcell*^®^* at 5 °C (71.8 ± 11.88%), BIOXcell*^®^* at RT (68.5 ± 17.35%), and INRA96*^®^* at RT (70.3 ± 13.46%). A marked reduction was observed at 24 h, with the lecithin-based extenders at 5 °C preserving progressive motility better than INRA96*^®^*.

**Figure 4:**
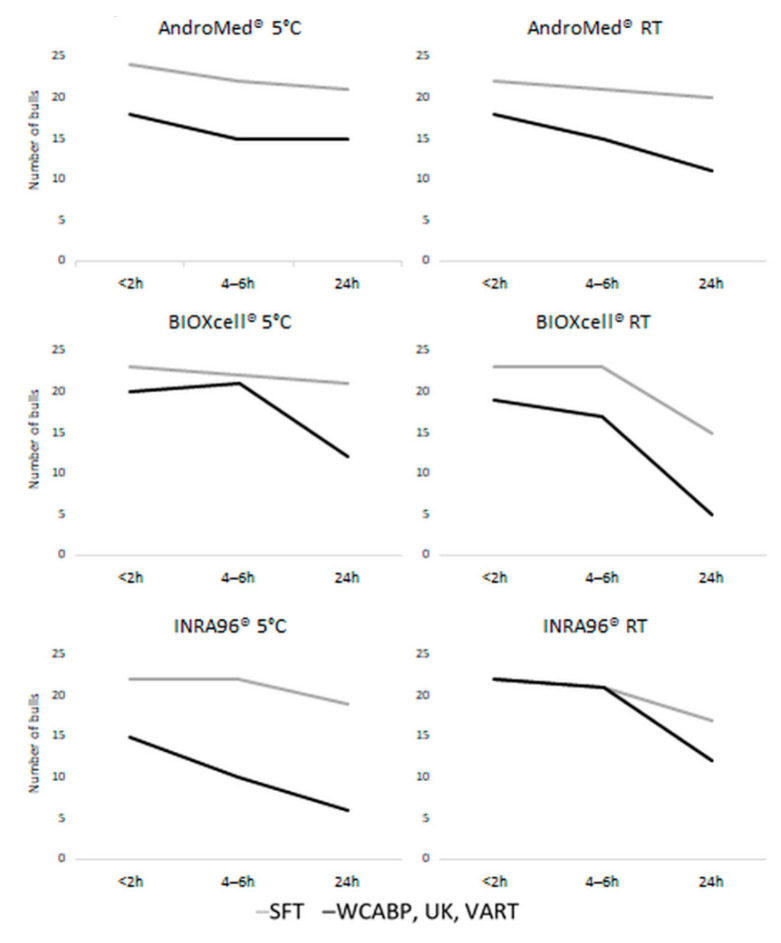
Number of bulls qualified as “suitable” according to different bull breeding soundness evaluation (BBSE) methods. SFT, Society For Theriogenology; UK; VART, Spanish guidelines “Valoración de la Aptitud Reproductiva de Sementales” (“Evaluation of Bull Reproductive Ability”). Abbreviations: A: AndroMed*^®^*; B: BIOXcell*^®^*; I: INRA96*^®^*; RT: room temperature; 5: refrigeration at 5 °C.

In general, the BBSE guidelines take into account only sperm morphology and sperm motility. In the BBSE recommended by the SFT, >70% normal spermatozoa and >30% progressive sperm motility are the cut-off values [9], whereas several other guidelines apply a cut-off of >60% for progressive motility [13]. In Figure 4, the percentages of bulls qualified as suitable according to various BBSE guidelines are shown. At the first evaluation, more than 85% of the bulls qualified as ‘suitable’ according to the SFT system, regardless of extender and storage temperature.

**Figure 5:**
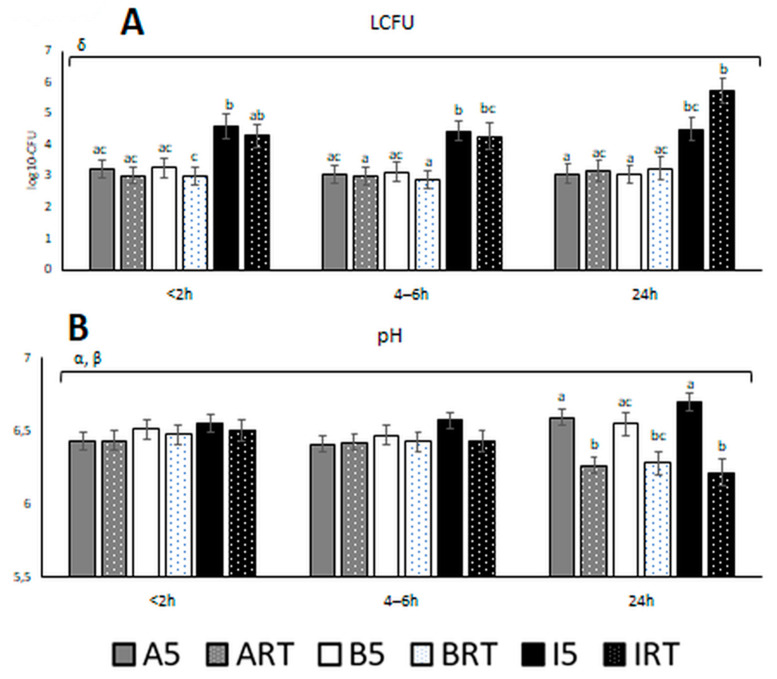
(**A**) Microbiological quality (log10-CFU) and (**B**) pH of bull semen at three different assessment time windows after semen collection, using three different semen extenders and two holding temperatures. Aliquots abbreviations: A: AndroMed*^®^*; B: BIOXcell*^®^*; I: INRA96*^®^*; RT: room temperature; 5: refrigeration at 5 °C. Factors abbreviations: t: time; Temp: temperature; Ex: extender; α: interaction temperature by extender; β: interaction temperature by time; γ: interaction extender by time; δ: interaction temperature by extender by time. Bars with different letters denote significantly different values (*p* < 0.05) among the six aliquots of the three extenders by two holding temperatures at each fixed time window (ANOVA). Bars without letters were not significantly different. Square bracket over the bars highlights significant effect (*p* < 0.05; from GLM analyses) of the factor or factors interactions during the time referred to with Greek letters. The bracket covers the time window where the effect is significant (from <2 to 4–6 h, from 4–6 to 24 h, or <2 to 24 h).

Analysis of log10-transformed CFUs revealed a triple interaction among temperature, extender, and time (*p* = 0.029), which means that microbiological growth was differently affected by each combination. Globally, the extender INRA96*^®^* was significantly less effective than AndroMed*^®^* and BIOXcell*^®^* for all storage durations, and INRA96*^®^* at RT allowed exponential bacterial growth between 4–6 and 24 h, which did not occur with AndroMed*^®^* or BIOXcell*^®^* (Figure 5). Both AndroMed*^®^* and BIOXcell*^®^* kept bacterial growth under control and similar at both temperatures. At the last time point (24 h), both extenders worked better at 5 °C than at RT (Figure 5, Table 1).

The values of pH were significantly affected by the double interactions temperature by extender (*p* = 0.02) and temperature by time (*p* < 0.001). The mean variation of pH with time occurred from 4–6 h up to 24 h after semen collection, with extenders at 5 °C inducing a slight basification of the samples (6.5 ± 0.26, 6.5 ± 0.38, and 6.7 ± 0.31 for AndroMed*^®^*, BIOXcell*^®^*, and INRA96*^®^*, respectively), and extenders kept at RT inducing a clear acidification (6.3 ± 0.28, 6.3 ± 0.39, and 6.2 ± 0.44 for AndroMed*^®^*, BIOXcell*^®^*, and INRA96*^®^*, respectively), with similar values among the three extenders. The smallest variation of pH over time was achieved by BIOXcell*^®^* at 5 °C (Figure 5).

### 3.2. Correlations of Semen Parameters with One Another and with Themselves over Time

All parameters in this study correlated with themselves across the three storage durations, with *r*-values >0.4 (*p* < 0.05). A general consistency among parameters was observed, and total motility correlated with progressive motility (Figure 3). Similarly, sperm vitality and normal morphology correlated positively with motility at all storage durations. Microbiological growth at the shortest storage duration correlated negatively with sperm vitality at longer storage durations (Figure 4 and Table 2).

## 4. Discussion

We conducted a study to evaluate the parameters affecting bull semen quality during short-term storage. Our results showed that the quality of bovine semen, assessed in terms of viability, morphology, motility, pH, and microbiological quality, was preserved up to 4–6 h post-ejaculation, independently of the extender used or the holding temperature. The only exception was the extender INRA96*^®^* at 5 °C, which led to the worst values in terms of sperm viability, motility, and microbiological load even at <2 h after collection. However, sperm motility decreased for all extenders and temperatures from 4–6 h to 24 h, with BIOXcell*^®^* at 5 °C giving the best values. Moreover, an acidification of the semen samples was observed from 4–6 h to 24 h in aliquots preserved at RT for all three extenders, while the microbiological load showed a dramatic increase with INRA96*^®^*.

Extenders provide nutrients and a stable medium where spermatozoa can survive for 60–72 h [35] or even 96 h [23] for subsequent artificial insemination. However, a decrease in total and progressive motility by time and temperature has been previously described during short-term storage of Holstein bull [23]. We observed lower motility at time <2 h than that study, and a sharper decrease in motility over time; this difference could be explained by differences between breeds and between artificial vagina and electroejaculation methods to collect semen. Ejaculates collected by artificial vagina have greater sperm motility, acrosome integrity, percentage of spermatozoa with active mitochondria, and lower DNA fragmentation than those collected by electroejaculation [36], probably indicating a physiological seminal sample.

Regardless of holding temperature, both sperm motility and fertility decline with time [23,35]. Consistently, we found that total and progressive motility decreased with time. With refrigerated samples, we observed better motility with soy lecithin-based extenders than with INRA96*^®^*. In contrast, a previous study of semen samples stored for up to 24 h at 15 °C observed adequate motility with INRA96*^®^* [23]. This difference may be due to the fact that penetrating cryoprotectants are not necessary at 15 °C, owing to their toxicity [37]. In agreement with other studies [22,23], we observed that INRA96*^®^* induced better viability, morphology, and motility values at RT than at 5 °C. Refrigeration induces cold shock injuries in sperm samples, damaging membrane integrity [15], which explains the need for cryoprotectants at 5 °C [25]. INRA96*^®^*, which worked less effectively than the other extenders at 5 °C, but not at RT, is formulated using caseins without glycerol. AndroMed*^®^* and BIOXcell*^®^* [38,39] contain phospholipids, soybean lecithin, and glycerol as cryoprotectants; hence, they work better below 14 °C, but not at a higher temperature [25,29]. Glycerol can protect spermatozoa from the age-related decline in fertility during inseminations involving fresh semen [40,41]. However, over 5 °C, it transiently increases osmotic pressure, which can reduce rates of pregnancy after artificial insemination [42].

Although extenders preserve sperm motility even up to three days after collection [23,43], insemination with fresh semen kept longer than 48 h is associated with lower conception rates than insemination with previously frozen semen [44]. The ability of sperm to migrate through artificial mucus in vitro is weakened after 48 h [45], and the causes may relate to motility, time window, extender, viability, the individual bull, and other factors.

The semen samples in our study showed adequate progressive motility enough to achieve pregnancy even 24 h after collection [22]. We observed a positive correlation between total and progressive motility and viability and morphology, which supports the idea that higher sperm motility goes hand-in-hand with higher fertility.

Viability and acrosomal integrity appear to be crucial for predicting bull fertility [46,47,48]. In our hands, sperm viability and live acrosome—intact spermatozoa decreased with time after 4–6 h, in agreement with a previous study [40]. Up to 24 h, the effectivity of AndroMed*^®^* and BIOXcell*^®^* in preserving viability depended on the holding temperature, with AndroMed*^®^* preserving sperm viability better at RT, while BIOXcell*^®^* performed better at 5 °C. Similarly, in the live acrosome—intact subpopulation, BIOXcell*^®^* was the best extender, especially after 24 h. Th different components of the extenders may explain our observations. In fact, a synergistic interaction of soybean lecithin with other components of the extenders has been proposed [49]. In ram semen studies, BIOXcell*^®^* showed a higher antioxidant activity than AndroMed*^®^* [50,51], leading to lower oxidative stress and t genomic damage in cryopreserved spermatozoa [52,53]. Another study showed that BIOXcell*^®^* preserved chromatin stability in sperm better than AndroMed*^®^* [53]. Extenders, temperatures, preparation procedures, or even holding length can affect semen parameters, as demonstrated in other studies [49,54,55,56].

Semen collected at Semen Stations inevitably contains ambient microorganisms, up to 2.36 × 104 ± 1943 CFU/mL in fresh semen [57] and 1.00 × 10 ± 90 CFU/mL in frozen semen after thawing [58]. Other authors found 103–106 CFU/mL in five bulls analysed after collection at an insemination centre [59] and 50.38 ± 16.29 CFU/mL in thawed cryopreserved bull semen collected under field conditions [60]. A recent study [61] isolated 135 microorganisms from 25 genera from 174 frozen–thawed semen collected from artificial vaginas and analysed at different periods of the conservation process. However, there is scarce information about the microbiological quality of semen collected in the field. The microbiological load of the samples analysed in our study was high even at <2 h, which likely reflects the field conditions, i.e.*,* commercial bulls electroejaculated on farms.

Interestingly, in our study, samples extended with INRA96*^®^* showed higher CFU counts than samples with other extenders, even at the shortest storage. Furthermore, microbiological load in INRA96*^®^* samples increased dramatically from 4–6 h to 24 h, especially when kept at RT. In contrast, BIOXcell*^®^* and AndroMed*^®^* kept the microbiological load stable over time, independently of temperature. This may reflect that AndroMed*^®^* and BIOXcell*^®^* incorporate tylosin, gentamicin, spectinomycin, and lincomycin, in accordance with European Commission (EC) Directive 88/407; INRA96*^®^*; however, they contain gentamicin, penicillin, and amphotericin-B. Our results suggest that INRA96*^®^* lacks the antibiotic efficacy to cope with the microbiological load of semen collected in the field. Unfortunately, we cannot compare our findings with other studies of INRA96*^®^* [23,44], as those works did not assess microbiological quality. Extenders may differ in their ability to bypass bacterial resistance. In one study [61], all 55 microorganisms evaluated were resistant to penicillin, contained in INRA96*^®^*, and all but one microorganism were resistant to tylosin and lincomycin, both present in BIOXcell*^®^* and AndroMed*^®^*. In a study of bull semen, resistance to all tested antibiotics was observed in 22% of all isolates from 135 different microorganisms, whereas only 3.9% of the isolates were inhibited by the antibiotics stipulated in EC Directive 88/407 [61]. A study on the bacterial load in frozen bull semen found that Gram-negative and -positive bacteria behaved differently in response to different antibiotics [62]. The most common flora in bull semen include Mycoplasma spp., Proteus spp., and Corynebacterium spp., while some other uncommon species, such as Pseudomonas aeruginosa, have been identified [59]. New antimicrobials such as fluorinate carboxyquinolone ofloxacin or ceftiofur/tylosin have begun to be tested in extenders [63].

The relatively high load in our field ejaculates may explain why our semen samples showed lower seminal parameters than in other studies [9,10,11,12,13]. CFU correlates negatively with sperm motility, viability, and morphology [60]. Similarly, we observed a negative time-dependent correlation between CFU and viability. Therefore, it is important to maximise hygiene standards during bull electroejaculation, and INRA96*^®^* does not seem to be an adequate extender for these conditions. INRA96*^®^* is registered as an extender for equine semen [32], collected under different conditions than bull ejaculates [64].

pH varied from 4–6 h to 24 h depending on temperature, becoming more acidic at RT and more basic at 5 °C. A pH between 7 and 7.5 has been identified as optimal for sperm [65]. Acidic pH inhibits the metabolic activity of spermatozoa, leading to accumulation of lactic acid, which reduces their motility [35,66]. Nevertheless, spermatozoa can tolerate a pH decrease to 5.5, but pH values below 5.5 are spermicidal [35]. In our study, pH at 5 °C never exceeded 6.70, whereas the pH at RT eventually fell to 6.22–6.28. This suggests that semen samples requiring storage for up to 24 h should be refrigerated.

Semen quality parameters in our study correlated with themselves over time, and the parameters at the shortest storage correlated more strongly with parameters at 4–6 h than with those at 24 h. This supports the idea that bull semen evaluation at 24 h after collection can reliably assess its quality, albeit with some underestimation.

We observed positive correlations of motility with viability and morphology. These findings support the previously documented interrelations [46,67]. Percentages of live acrosome—intact sperm positively correlated with viability, with this correlation between sperm viability and membranes previously described [33,34,49]. The bacterial load in our study correlated negatively with viability, which suggests a negative impact of microbiological contamination.

We found that the percentage of bulls in our study that would be considered “suitable” based on BBSE standards depended on holding temperature, time, and type of extender used for short-term storage. Therefore, practitioners should consider these factors during evaluation in order to optimize semen quality as much as possible and avoid undervaluing bulls.

## 5. Conclusions

The quality of bovine semen, as assessed in terms of viability, morphology, motility, pH, and microbiological quality, can be preserved for up to 6 h after collection at 5 °C or RT in the presence of the extenders AndroMed*^®^* or BIOXcell*^®^*, or at RT in the presence of the extender INRA96*^®^*. If semen samples should be kept for 6–24 h, soy lecithin-based extenders and refrigeration should be used.

## Figures and Tables

**Table 1:** Colony-forming units (CFUs) for semen samples, stratified by extender, storage duration, and storage temperature.

**Extender**	**<2 h**	
	**5 °C**	**Room Temperature**
AndroMed^®^	34,005 ^a,c^ ± 68,899	19,923 ^a,c^ ± 46,652
BIOXcell^®^	145,451 ^a,c^ ± 597,538	121,654 ^c^ ± 558,367
INRA96^®^	25,466,834 ^b^ ± 82,941,641	21,824,412 ^a, b^ ± 73,257,122
**Extender**	**4–6 h**	
	**5 °C**	**Room Temperature**
AndroMed^®^	23,906 ^a,c^ ± 42,218	19,366 ^a^ ± 56,645
BIOXcell^®^	160,113 ^a,c^ ± 676,803	31,612 ^a^ ± 104,373
INRA96^®^	5,692,810 ^b^ ± 24,774,913	26,230,196 ^b, c^ ± 99,887,451
**Extender**	**24 h**	
	**5 °C**	**Room Temperature**
AndroMed^®^	60,572 ^a^ ± 205,292	71,884 ^a, c^ ± 151,384
BIOXcell^®^	32,566 ^a^ ± 83,193	101,347 ^a,c^ ± 201,840
INRA96^®^	13,386,748 ^b,c^ ± 49,276,426	181,508,58 ^b^ ± 488,516,056

Results are expressed in CFU/mL. Figures with different superscript letters denote significantly different values (*p* < 0.05) among the six aliquots of the three extenders by two holding temperatures at each fixed time window (ANOVA).

**Table 2:** Pearson coefficients (r) for correlations between bull semen quality parameters determined after three durations of storage in the presence of three semen extenders at two holding temperatures.

Parameters	logCFU-T4	logCFU-T24	PMot-T < 2	PMot-T4	PMot-T24	SpV-T < 2	SpV-T4	SpV-T24	SpM-T < 2	SpM-T4	SpM-T24
TMot-T < 2			0.947	0.707	0.481	0.607	0.688	0.605	0.524	0.518	0.472
TMot-T4				0.909	0.517		0.69	0.62		0.518	0.485
TMot-T24					0.855			0.602			
PMot-T < 2						0.512	0.622	0.544	0.533	0.538	0.472
PMot-T4							0.569	0.496		0.489	0.448
PMot-T24								0.467			
logCFU-T < 2							−0.42	−0.401			
logCFU-T4							−0.405				
LAcI-T < 2						0.433		0.411			
LAcI-T4					0.405		0.428				
LAcI-T24								0.54			

Only statistically significant correlations (*p* < 0.05) are shown. Abbreviations: logCFU: log10-transformed colony-forming units; SpV: sperm viability; SpM: sperm morphology; TMot: total motility; PMot: progressive motility; LAcI: live acrosome-intact; T < 2: time <2 h; T4: time 4–6 h; T24: time 24 h.

## Data Availability

Data are contained within the article. Raw data are available on request by the authors.
